# Current Insights in the Development of Efficacious Vaccines Against RSV

**DOI:** 10.3389/fimmu.2020.01507

**Published:** 2020-07-17

**Authors:** Jorge A. Soto, Laura M. Stephens, Kody A. Waldstein, Gisela Canedo-Marroquín, Steven M. Varga, Alexis M. Kalergis

**Affiliations:** ^1^Departamento de Genética Molecular y Microbiología, Facultad de Ciencias Biológicas, Instituto Milenio de Inmunología e Inmunoterapia, Pontificia Universidad Católica de Chile, Santiago, Chile; ^2^Interdisciplinary Graduate Program in Immunology, University of Iowa, Iowa City, IA, United States; ^3^Department of Microbiology and Immunology, University of Iowa, Iowa City, IA, United States; ^4^Department of Pathology, University of Iowa, Iowa City, IA, United States; ^5^Departamento de Endocrinología, Facultad de Medicina, Instituto Milenio de Inmunología e Inmunoterapia, Pontificia Universidad Católica de Chile, Santiago, Chile

**Keywords:** Respiratory Syncytial Virus, treatments, vaccines, antibodies, T cells

## Introduction

Respiratory viral infections are one of the most important global public health burdens, resulting in millions of hospitalizations worldwide annually (1, 2). Respiratory Syncytial Virus (RSV) is the leading cause of acute lower respiratory tract infections (ALRTI) in children under the age of 2 (3) and adults over 65 (4). RSV-induced disease can range from symptoms similar to the common cold to complex respiratory diseases, such as pneumonia or bronchiolitis, leading to extrapulmonary sequelae in the brain and other tissues (5). During the 1960s, a formalin-inactivated RSV (FI-RSV) vaccine was evaluated in children. Vaccinated individuals exhibited increased disease severity upon subsequent natural RSV infection compared to the controls (6–9). This vaccine-enhanced disease resulted from the failure of the vaccine to elicit either potent neutralizing antibodies or memory CD8^+^ T cells as well as the induction of a strong inflammatory CD4 T cell response (10–13). Currently, the only treatment option available for RSV is a humanized monoclonal antibody against the RSV F surface protein, known as palivizumab (14). However, its usage is limited to high-risk individuals, such as preterm babies, and infants with congenic diseases (15–17). Due to prolonged concerns about vaccine safety, a better understanding of RSV-induced pathogenesis and the host immune response is needed to aid in the development of safe and effective treatments and vaccines for RSV. This Opinion article examines the various vaccine modalities currently undergoing testing and discusses the advantages and disadvantages of the strategies being employed.

## RSV Vaccine Modalities and Lessons From the Host Immune Response

Based on the knowledge gained from the unsuccessful FI-RSV vaccine trial, new vaccine formulations are being developed that promote neutralizing antibodies, induce activated memory and lung-resident CD8^+^ T cells, and can be administered to different target populations including children, elderly and pregnant women. The most promising vaccine candidates currently being evaluated in humans are live-attenuated, recombinant vector-based, and subunit vaccines.

Live-attenuated vaccines demonstrate favorable benefits including a low risk of causing vaccine-enhanced disease, and they can promote both a humoral and cellular immune response. However, potential drawbacks include conserving the stability of the formulation, and balancing the attenuation of the virus while maintaining replicative activity and immunogenicity in the host (18). Additionally, further studies are needed to assess the safety of live-attenuated vaccines in multiple populations (19). Many live-attenuated vaccines are currently undergoing testing in clinical trials and demonstrate a robust induction of a humoral immune response; however, much less information is known about the cellular immune responses induced by the vaccines. Deletion of the M2-2 protein from the RSV strain A2 (LIDΔM2-2) induced robust serum RSV-specific IgG and neutralizing antibody titers that correlated with lower nasal wash viral titers administered intranasally to seronegative children (20). A similar induction of serum neutralizing antibodies was observed with a cold-passage/stabilized RSV containing several attenuating point mutations as well as deletion of the small hydrophobic (SH) protein (RSVcps2) (21). Finally, preclinical studies of a recombinant BCG vaccine expressing the RSV nucleoprotein (N) demonstrated an effective cellular and humoral immune response in mice (22–25).

Recombinant vector-based vaccines allow the presentation of one or more antigens expressed on a viral vector such as parainfluenza virus type 3 (PIV3) or adenovirus. This allows for natural presentation of the antigen of interest to immune cells. A PIV3 vector expressing the RSV F protein (MEDI-534) demonstrated safety in a Phase 1 study when administered intranasally to young children (26). Interestingly, some discordance was observed in the specificity of the immune response. Sequencing of viral samples suggested that modifications were generated post-vaccination in a number of subjects that promoted a reduction in the expression of the F protein correlating with lower neutralizing antibodies in those individuals (27). A recently developed vaccine composed of a chimpanzee adenovirus viral vector expressing the RSV F, N, and M2-1 proteins (ChAd155-RSV) induced robust neutralizing antibody titers and interferon gamma (IFNγ)-secreting T cells compared to placebo controls (28). ReiThera Srl developed a similar vaccine using a chimpanzee adenovirus (PanAd3) viral vector expressing the RSV F, N, and M2-1 proteins in combination with a modified vaccinia virus Ankara (MVA). Intramuscular and intranasal delivery of the vaccine to healthy adults was well tolerated and induced both RSV-specific antibody titers and RSV-specific CD4 and CD8^+^ T cells (29–31). Interestingly, other clinical trials using adenovirus have been developed to date (NCT03982199, NCT03636906, among others).

Subunit vaccines are a common vaccine modality; however, some disadvantages are associated with these formulations such as the frequent need to use an adjuvant to increase the immunogenicity. A single dose of an RSV F protein subunit vaccine combined with aluminum hydroxide induced RSV F-specific antibodies that persisted for >180 days post-vaccination (32). Similar results were observed following intramuscular administration to women of child-bearing age, suggesting that maternal immunization with this vaccine candidate could generate lasting antibodies to passively transfer to the fetus during the pregnancy (33). Another vaccine utilizing the RSV F protein demonstrated safety and efficacy in Phase 1 and Phase 2 clinicals trials when administered without an adjuvant, suggesting that a subunit vaccine may induce lasting protection without an added adjuvant (34). Interestingly, formulations using the RSV F protein have failed to provide protection against RSV infection in older adult populations, indicating that subunit vaccines may not be the best candidate for this target population (35).

Many vaccine prototypes are focused on viral surface proteins (36, 37). One of the most common viral targets for antibodies is the RSV fusion (F) protein (38, 39). Vaccine formulations containing the N protein also induce long-lasting neutralizing antibodies and could serve as a novel antiviral target (22, 25). The RSV G protein is involved in the initiation of the virus life cycle and has a potent effect on the regulation of the immune response (36). The SH protein can promote a protective immune response in animal models of RSV through Fc receptor-mediated interactions with macrophages and helping the promotion of long-lasting antibodies (40, 41). Furthermore, other protein targets are currently being or have been evaluated in clinical trials, including the nonstructural protein 2 (NS2) (NCT03596801, NCT03473002) and the M2-2 protein (20, 42). However, independent of the antigen evaluated, the key requirement of any RSV vaccine is the ability to promote a safe, but effective and protective immune response.

On the other hand, another type of vaccine strategy that has provided positive and interesting results in human tests is based on intranasal administration of a novel BLP (bacterium like particle) conjugated to the RSV fusion (F) protein eliciting both mucosal IgA responses and elevated IFN-γ production (43). Since BLP prototype is a promising strategy, more assays to evaluate long-lasting immune response are required.

The choice of administration route is an important decision in vaccine development, with most vaccines being delivered via the sublingual, intramuscular, or intranasal route. Sublingual administration of an RSV G protein vaccine induced enhanced cellular infiltration and pro-inflammatory cytokine production compared to intranasal delivery (37). Similar results were observed when a recombinant RSV attachment (G) protein containing the central regions for both RSV A and B serotypes was administrated either intranasally or sublingually (44). Sublingual delivery enhanced pulmonary eosinophil recruitment and body weight loss, while intranasal administration promoted enhanced IgG and IgA antibodies and lower pro-inflammatory cell recruitment into the lung. Mucosal administration may also induce a high titer of IgA in bronchial alveolar lavage (BAL) fluid and IgG antibodies in serum (44, 45). A murine cytomegalovirus vector expressing the RSV matrix (M) protein induced robust lung-resident memory T cell populations when administered intranasally compared to intraperitoneally, where this population was almost undetectable (46, 47). This suggests that intranasal administration of an RSV vaccine would induce an enhanced CD8^+^ T cell response, a strong secretion of IgG and IgA antibodies, and decrease the inflammatory state of the lung.

One way to aid in the successful development of an RSV vaccine is to gain a better understanding of the host immune response to the virus and the factors required for long-term immunity. Studies examining the host response during acute infection of infants suggest that the virus elicits a pathogenic Th2 dominant response (10–13). Th2-biased T cells, driven by IL-4, IL-5, and IL-13 cytokines, lead to inflammation and hyperreactivity of the airways (48–51). Other T cell populations, including regulatory T cells (Tregs) and Th17 cells, also play an important role during RSV infection (52). Th17 cells can promote a pro-inflammatory state leading to enhanced neutrophil recruitment and reduced CD8^+^ T cell activation (53). Tregs are associated with the active recruitment of cytotoxic CD8^+^ T cells in the lung; however, unbalanced Tregs could promote enhanced lung damage (54, 55). Interestingly, peripheral blood mononuclear cells (PBMCs) from infected children exhibit reduced Tregs compared to age-matched controls (56). Similarly, depletion of Tregs in mice promoted enhanced lung pathology following RSV infection (57, 58). Thus, a successful vaccine should induce a balanced T cell response characterized by Th1-biased T cells as well as Tregs.

The induction of type I IFN are essential for RSV viral clearance. The absence of type I IFN promotes a pro-inflammatory response that helps to induce a lung pathology in both human and murine models of infection (59). The administration of IFN-α in RSV-naïve high-risk infants is associated with a decrease in lung pathology and enhanced viral clearance. However, RSV possesses several evasion mechanisms, and both the NS1 protein and the G protein can suppress the type I IFN response (60). A vaccine that induces a powerful type I IFN secretion within its response could be considered a good candidate against RSV.

CD8^+^ T cells play a critical role in RSV-clearance (61). Murine studies of RSV demonstrate a protective role for memory CD8^+^ T cells in promoting viral clearance and providing protection from reinfection (61, 62). Nevertheless, natural RSV infection induces low levels of CD8^+^ T cells. Thus, it would be advantageous for a vaccine to promote a Th-1 immune response and generate memory CD8^+^ T cells (23–25). In contrast, CD4^+^ T cells have a controversial role during RSV infection. Following natural infection, CD4^+^ T cells can promote a dysbalanced host response that enhances immunopathology. However, adoptive transfer studies in the mouse model also suggest that CD4^+^ T cells can play a protective role. The induction of a Th-1 polarized immune response that promotes both CD8^+^ and CD4^+^ T cells is essential for a vaccine to induce a protective immune response against RSV.

The decline in neutralizing antibodies after the RSV infection is an important factor in the reinfections that occur in children. Several formulations of vaccines seek to induce neutralizing antibodies in high risk populations and maternal antibodies that will be transferred from the mother to the fetus to protect against early RSV infections. Nevertheless, these formulations have been shown to induce antibodies that are short-lived. Interestingly, intranasal vaccines have demonstrated the ability to induce high levels of neutralizing antibodies and also promote the IgA secretion that is directly associated with a protective immune response against RSV (43–45, 59, 63).

## Discussion

Severe RSV-induced disease continues to present a major global health burden in high-risk groups such as preterm infants, newborns, elderly populations, and those with many associated comorbidities. There is no licensed vaccine to prevent RSV infections, and the only prophylaxis currently approved by the Food and Drug Administration (FDA) is the monoclonal antibody palivizumab. However, its limited use in high-risk groups (14), as well as the high cost and moderate effectiveness underscore the need for additional options. There remains a critical need to develop safe and effective RSV vaccines and therapeutics to combat RSV disease severity in infants and high-risk populations.

In conclusion a vaccine against RSV that promotes an effective antiviral response must induce a prolonged neutralizing antibody response, Th-1 polarized immunity that promotes both CD8^+^ and CD4^+^ T cells, type I IFN secretion and an efficient mucosa immune response.

## Author Contributions

JS, LS, and KW wrote and revised the manuscript. GC-M contributed to the revision and editing of the manuscript. SV and AK were the lead investigators and revised the manuscript. All authors contributed to the article and approved the submitted version.

## Conflict of Interest

The authors declare that the research was conducted in the absence of any commercial or financial relationships that could be construed as a potential conflict of interest.
